# Clinicopathological significance of mitochondrial D-Loop mutations in head and neck carcinoma

**DOI:** 10.1038/sj.bjc.6602993

**Published:** 2006-02-21

**Authors:** A Lièvre, H Blons, A M Houllier, O Laccourreye, D Brasnu, P Beaune, P Laurent-Puig

**Affiliations:** 1 INSERM, U490, Université René Descartes, Paris F-75006, France; 2 Assistance Publique-Hôpitaux de Paris, Hôpital Européen Georges Pompidou, pôle biologie, Paris F-75015, France; 3 Assistance Publique-Hôpitaux de Paris, Hôpital Européen Georges Pompidou, service d'Oto-Rhino-Laryngologie et de Chirurgie cervico-faciale, Paris F-75015, France

**Keywords:** mitochondrial DNA, mutation, head and neck cancer, tobacco, chemotherapy, prognosis

## Abstract

Mitochondrial DNA mutations have been reported in several types of tumours, including head and neck squamous cell carcinoma (HNSCC). The noncoding region of the Displacement-Loop (D-Loop) has emerged as a mutational hotspot and we recently found that they were associated with prognosis and response to 5 fluorouracil (5FU) in colon cancers. In order to evaluate the frequence of D-Loop mutations in a large series of HNSCC and establish correlations with clinicopathologic parameters, we sequenced the D-Loop of 109 HNSCC before a treatment by neoadjuvant 5FU-cisplatin-based chemotherapy and surgery. Then, we correlated these mutations with prognosis and response to chemotherapy. A D-Loop mutation was identified in 21% of the tumors, the majority of them were located in a C-tract (D310). The prevalence of D310 mutations increased significantly with the number of cytosines in the matched normal tissue sequence (*P*=0.02). Hypopharyngeal cancer was significantly more frequent (*P*=0.03) and tobacco consumption more important (*P*=0.01) in the group of patients with D-Loop mutation. The presence of D-Loop mutation was not associated with prognosis or with response to neoadjuvant chemotherapy. These results suggest that D-Loop mutations should be considered as a cancer biomarker that may be useful for the early detection of HNSCC in individuals at risk of this cancer.

Although much knowledge has been collected concerning alterations in cancer cell nuclear DNA (nDNA), less attention has been paid to mutations within mitochondrial DNA (mtDNA). Mitochondrial DNA is a 16 569 bp double-stranded, circular DNA encoding 13 respiratory chain protein subunits, 22 tRNAs and two rRNAs. It is also composed of a 1.2 kb noncoding region, the Displacement-Loop (D-Loop), which contains essential transcription and replication elements. Owing to a particular susceptibility to oxidative damage due to high levels of reactive oxygen species (ROS) generation in mitochondria, inefficient DNA repair system and a lack of protective histones in this organelle, mutation rate has been reported to be 10–17-fold higher in the mtDNA than in the nDNA (Fliss *et al*, 2000). Mitochondria has long been suspected to be involved in carcinogenesis (Warburg, 1956). Its role in apoptosis supports this hypothesis (Zamzami and Kroemer, 2001) and it was shown that mtDNA mutations may lead to a dysregulation of oxidative phosphorylation that can enhance production of the carcinogenic ROS. Over last years, somatic mtDNA mutations have been reported in many human tumours (Polyak *et al*, 1998; Maximo *et al*, 2000; Richard *et al*, 2000; Yeh *et al*, 2000; Hibi *et al*, 2001b; Sanchez-Cespedes *et al*, 2001; Kirches *et al*, 2001; Liu *et al*, 2001; Lievre *et al*, 2005), including head and neck squamous cell carcinoma (HNSCC) that were found mutated in small series in 37–77% of the cases (Fliss *et al*, 2000; Sanchez-Cespedes *et al*, 2001; Ha *et al*, 2002; Tan *et al*, 2003; Poetsch *et al*, 2004). Although mutations may occur throughout the mitochondrial genome, the vast majority of them have been described in the noncoding region of the D-Loop and particularly in a mononucleotide repeat named D310 (C-tract, nucleotide position: 303–315) that has emerged as a mutational hotspot in HNSCC (Fliss *et al*, 2000; Sanchez-Cespedes *et al*, 2001; Ha *et al*, 2002; Tan *et al*, 2003; Poetsch *et al*, 2004).

Head and neck squamous cell carcinoma represents 5% of all newly diagnosed cancer cases in the northern and western Europe and in the US (Muir and Weiland, 1995) where it represents a public health problem. As in most solid tumours, head and neck tumor epithelial cells undergo nuclear genetic alterations in proto-oncogenes and tumour suppressor genes through a multistep process. One of the most frequent alterations are *TP53* somatic mutations found in more than a half of the cases. Allelic losses are also frequently observed on 3p, 9p and 17p (Nawroz *et al*, 1994; van der Riet *et al*, 1994; Blons *et al*, 1999), as the amplification of the cyclin D1 oncogene. Identification of new genetic alterations associated with HNSCC is important since they may allow to better understand the molecular mechanisms involved in head and neck carcinogenesis and serve as a molecular marker that may be used in evaluating the tumorigenic potential of head and neck lesions in individuals at high risk of cancer. Recently, we found that D-Loop mutations were linked to prognosis and lack of benefit from 5 fluorouracil (5FU)-based adjuvant chemotherapy in colorectal carcinomas (Lievre *et al*, 2005). Neoadjuvant chemotherapy have been recently developed in new treatment strategies of locally advanced HNSCC and has been shown to be curative in complete clinical responder patients with a cancer of the pharyngolarynx (Laccourreye *et al*, 2001). Identification of molecular factors associated with response to neoadjuvant chemotherapy has become an important goal because it may help to select patients who could benefit from this treatment and so from a possible organ preservation. Therefore, the aims of this work were to determine the frequency of D-Loop mutations in a large series of HNSCC, establish correlations between D-Loop mutations and clinicopathologic parameters and determine the impact of these mutations on prognosis and response to neoadjuvant 5FU–cisplatin-based chemotherapy in HNSCC patients.

## MATERIALS AND METHODS

### Patients

This study was performed on patients with histologically proven HNSCC managed at the Laennec Hospital (Paris, France) who had been prospectively included in a previous study in which response to neoadjuvant chemotherapy was assessed (Blons *et al*, 2004). The inclusion criteria retained were the following: no previous history of cancer, no multiple tumour locations, no contraindication for a 5FU- or cisplatin-based chemotherapy and indication for neoadjuvant chemotherapy prior to surgery or radiotherapy. This work was performed according to the French Law and blood samples and tumour biopsies were obtained after written informed consent and approval of the local ethic committee (CCPPRB-#96,017). Among the 148 patients initially included, 109 (98 male and 11 female subjects, mean age: 57.8±1 years) for whom DNA were still available were screened for mtDNA mutations. Tumours were located in the oral cavity (*n*=13), the oropharynx (*n*=46), the hypopharynx (*n*=27) and the endolarynx (*n*=23). They were classified according to the TNM classification and staged as recommended by the American Joint Committee on Cancer. There were five T1, 42 T2, 28 T3, 34 T4 tumours, and 49 tumours were N0 whereas 60 of them were N+. Three tumours were stage I, 23 were stage II, 25 were stage III and 58 were stage IV. Among the patients, 63 smoked >35 pack-years; 34, 15–35 pack-years and 12, <15 pack-years. A *TP53* mutation was present in 72 patients (67.3%). Clinicopathologic characteristics of the patients are listed in Table 1. All patients received a neoadjuvant chemotherapy before surgery or radiotherapy that consisted of cisplatin (25 mg m^−2^ day^−1^) and 5FU (1 g m^−2^ day^−1^) delivered as a daily continuous i.v. dose in 4-day courses. Three courses were repeated at 16–21 days intervals. Clinical response was assessed as defined by the Eastern Cooperative Oncology Group. Responder patients (R) were defined by patients who showed at least a 50% decrease in tumour size and nonresponder patients (NR) by those who showed <50% decrease in tumour size. In this series, 72 patients (66.1%) were responders and 37 (33.9%) were nonresponders.

### Tissue sample preparation and DNA extraction

Tumour and 10 ml of blood from each patient were collected at the initial diagnosis during endoscopy under general anesthesia. All tumour samples were diagnosed as invasive squamous-cell carcinoma after a histopathological analysis and then frozen in liquid nitrogen. Lymphocytes and tumour tissues samples were stored at −80°C and extracted as described previously (Blons *et al*, 1999).

### Displacement-Loop amplification

Polymerase chain reaction (PCR) amplification of the D-Loop was performed on a Gen Amp PCR System 9700 (Applied Biosystems, Foster City, CA, USA) using primers F47: 5′-CGC ACG GAC TAC AAC CAC GAC-3′ (forward) and R15: 5′-CTG TGG GGG GTG TCT TTG GG (reverse) as described previously (Lievre *et al*, 2005). The 2467 bp PCR products (nucleotide positions: 14679–577) were then purified using G-50 Sephadex superfine (Amersham Biosciences, Orsay, France) on Multiscreen support (Millipore, Bedford, MA, USA).

### Direct sequencing of the D310 repeat

The D310 repeat sequencing was performed on a Gene Amp PCR System 9700 (Applied Biosystems) using a Big Dye Terminator cycle sequencing kit (Applied Biosystems) as described previously (Lievre *et al*, 2005). Sequences were analysed on an ABI Prism® 3900 DNA Analyser automated sequencer (Applied Biosystems). The results of DNA sequence analysis were compared with the published reference mtDNA sequence (GenBank, access number J01415) using Autoassembler® software (Applied Biosystems). A 400 bp fragment of the D-Loop (nucleotide position: 190–590) containing the D310 homopolymeric C-tract from each patient was analysed. Any mtDNA sequences that differed between tumour and matched lymphocytes mtDNA were scored as somatic mutations. All somatic mutations found were further validated by a new independent amplification and sequencing.

### Statistical analysis

The *χ*^2^-test was used to determine the relationship between each categorical variable and D-Loop mutations, and a *t*-test to determine the relationship between quantitative variables and D-Loop mutations. Survival curves were constructed using the Kaplan–Meier method and compared using the log-rank test. The median time of survival was used to summarise the survival data. These statistical tests were performed using the STATA software (STATA 7.0; College Station, TX, USA). A *P*-value <0.05 was used to indicate statistical significance.

## RESULTS

### Displacement-Loop mutations

Displacement-Loop sequence analysis was performed in 109 patients. A total of 25 somatic D-Loop mutations were identified in 23 of the 109 (21%) tumours (Table 2). The majority of the mutations were located in the D310 mononucleotide repeat (19 out of 25, 76%). These mutations were insertions or deletions of one (*n*=15) to several (*n*=4) base pairs. Six mutations were found outside the D310 sequence. Among them, five were substitution of one base pair. The last one was a CA deletion at the nucleotide position 514. Two patients (nos. 22 and 153) had two mutations: in each of them, a D310 mutation coexisted with a D-Loop mutation located outside the D310 repeat. Among all mutations, 10 (40%) were homoplasmic and 15 (60%) were heteroplasmic. Homoplasmic mutations were significantly more frequent in the group of D-Loop mutations located outside the D310 repeat (5/6) than in the group of D310 mutations (five out of 19) (83 *vs* 26%, *P*=0.02).

The D310 sequence is polymorphic in the human population. The number of cytosines in the 7-bp tract varied from 6 to 13 and the most frequent sequences for the D310 region are C_7_TC_6_, C_8_TC_6_ and C_9_TC_6_. In our series, the D310 sequence in nonmalignant tumour tissues was homoplasmic in 83.5% and heteoplasmic in 16.5% of the cases and was distributed as follows: C_7_TC_6_ in 65 out of 109 (59.6%), C_8_TC_6_ in 20 out of 109 (18.4%), C_9_TC_6_ in six out of 109 (5.5%), C_8_TC_6_/C_9_TC_6_ in 17 out of 109 (15.6%) and C_9_TC_6_/C_10_TC_6_ in one out of 109 (0.9%). The prevalence of tumour D310 mutations increased significantly with the number of cytosines in the matched normal tissue sequence. Indeed, the C_7_TC_6_ sequence was altered in eight patients out of the 65 (12%), the C_8_TC_6_ and C_8_TC_6_/C_9_TC_6_ sequences were altered in 12 patients out of the 37 (32%) and the C_9_TC_6_ and C_9_TC_6_/C_10_TC_6_ sequences were altered in three out of the seven cases (43%) (*P*=0.02).

A total of 24 germline polymorphisms were found (Table 3). Twenty-one were already reported in the MITOMAP database and three of them (A240G, A249G and C534T) are novel.

### Association of D-Loop mutations and clinicopathologic characteristics

The analysis of clinicopathologic variables showed a significant difference in tumour site between tumours with and without D-Loop mutation (*P*=0.03). Indeed, among D-Loop-mutated tumours, 47.8% were located in the hypopharynx as compared to 18.6% in the group of nonmutated tumours (Table 1). Moreover, presence of D-Loop mutation was significantly associated with tobacco consumption: the mean number of pack-year in the group of patients with tumour D-Loop mutation was 48±3 compared to 37±2 for patients without mutation (*P*=0.01). The analysis of other clinicopathologic variables showed no correlation between the presence of a tumour D-Loop mutation and, respectively, age, gender, tumour stage and *TP53* mutation (Table 1).

### Association of D-Loop mutations and response to chemotherapy and survival

Tumours were classified according to the decrease in tumour size after 5FU–cisplatin-based chemotherapy as it was defined in the Materials and Methods section. In all, 72 patients (66.1%) were responders and 37 (33.9%) were nonresponders. No correlation was found between the presence of tumour D-Loop mutation and response to neoadjuvant chemotherapy (Table 1).

**Figure 1:**
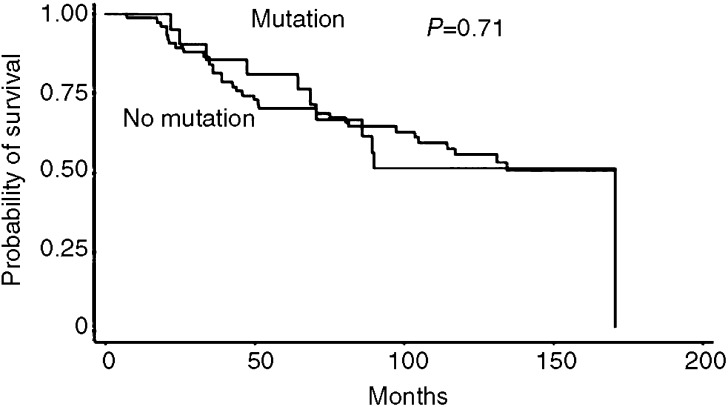
Overall survival curves of HNSCC patients with and without tumour D-Loop mutation.

The presence of D-Loop mutation was not a prognostic factor: the 5-year overall survival of patients with tumour D-Loop mutation was 81% compared to 70% for patients without mutation (*P*=0.71, Figure 1).

## DISCUSSION

In the past few years, somatic mtDNA mutations have been identified in several types of human tumours (Polyak *et al*, 1998; Fliss *et al*, 2000; Maximo *et al*, 2000; Richard *et al*, 2000; Yeh *et al*, 2000; Hibi *et al*, 2001b; Kirches *et al*, 2001; Liu *et al*, 2001; Sanchez-Cespedes *et al*, 2001; Nomoto *et al*, 2002; Lievre *et al*, 2005), including HNSCC (Fliss *et al*, 2000; Sanchez-Cespedes *et al*, 2001; Ha *et al*, 2002; Poetsch *et al*, 2004). In the present study, which is the largest series of HNSCC analysed for mtDNA mutations in the literature, we report a 21% frequency of D-Loop somatic mutations. This frequency is similar to that obtained by Fliss *et al* (2000) who performed a sequence analysis of 80% of the mitochondrial genome and found a D-Loop mutation in three of 13 (23%) HNSCC patients. However, other studies on small series of patients reported more frequent D-Loop mutations, ranging from 37 to 61% (Sanchez-Cespedes *et al*, 2001; Ha *et al*, 2002; Poetsch *et al*, 2004) of the cases. In these studies, D310 repeat emerged as a mutational hotspot. On 67 primary HNSCC from 56 patients, Poetsch *et al* (2004) sequenced two parts of the D-Loop and two mitochondrial genes (*MTND1* and *MTND5*). They found all mtDNA mutations in a part of the D-Loop and a mtDNA microsatellite instability, defined as insertions or deletions in the D310 repeat, in 42% of the tumours. Another study showed 66% of D-Loop mutations in 18 oral cancers of betel quid chewers (Tan *et al*, 2003), which however cannot be really assimilated to HNSCC of tobacco and alcohol consumers. The vast majority of these mutations were located between nucleotides 204 and 489 and 44% of the tumours harboured mutations in the D310 repeat. These results led us to focus our sequence analysis on a 400 bp fragment of the D-Loop (nucleotide position: 190–490) containing the D310 C-tract which was found to be a hotspot of mutations since more than 80% of the D-Loop mutations were located in this sequence, as it was suggested previously (Sanchez-Cespedes *et al*, 2001; Ha *et al*, 2002). The differences in D-Loop mutation frequency observed between our study, that of *Fliss et al* and the other studies remain to be explained. Overestimation of gene mutation rates is frequently observed in series of small sample size, which is the case of previous studies that have specifically addressed the D-Loop mutation frequency issue in HNSCC. As a consequence, our study gives new information as regards to the frequency of D-Loop mutations in HNSCC that has likely been overestimated in previous studies of small samples size. Another explanation may be differences in distribution of tumour sites between studies. We showed that tumours located in the hypopharynx (24.8% of all tumours) were significantly more mutated than tumours in other sites. Proportion of hypopharynx tumours is not known in the other studies (Fliss *et al*, 2000; Sanchez-Cespedes *et al*, 2001; Ha *et al*, 2002; Poetsch *et al*, 2004). So, we can speculate that a higher proportion of tumours located in the hypopharynx would be associated with a higher frequency of mtDNA mutation. Moreover, in the present study, the prevalence of tumour D310 mutations increased significantly with the number of cytosines in the D310 sequence of matched normal tissues. According to these findings, the frequency of D-Loop mutations may depend on the proportion of the different D310 sequences in the normal tissues (C_7−8−9−10_TC_6_), which is probably not the same in the different studies. Finally, the mtDNA mutagenesis process in oral squamous cell carcinoma of betel quid chewers is likely to be different to that of cigarettes smokers. Betel quid contains tender areca nuts and lime that have been shown to generate ROS and induce oxidative DNA damage (Nair *et al*, 1987; Stich and Anders, 1989), which can initiate or promote oral carcinogenesis. These betel quid compounds could therefore preferentially target mtDNA, which may explain the high proportion of oral tumours with mtDNA mutation observed in betel quid chewers (Tan *et al*, 2003), compared to that observed in cigarette smokers.

With a frequency of more than 20% in HNSCC, D-Loop mutations may be an interesting molecular marker in the evaluation of the tumorigenic potential of head and neck lesions in individuals at high risk of this cancer. They were shown to be an early event in head and neck carcinogenesis (Ha *et al*, 2002). Their frequence was 22% in the earliest head and neck premalignant lesions and increased with the degree of dysplasia to reach 50% in lesions of severe dysplasia and 61% in carcinomas *in situ* in a recent study (Ha *et al*, 2002). Similar results were obtained in prostate (Jeronimo *et al*, 2001) and oesophagus adenocarcinoma (Miyazono *et al*, 2002) where identical D-Loop mutations were found both in primary tumours and corresponding premalignant lesions, which is consistent with a process of clonal evolution. These data suggest that D-Loop mutations could be considered as a cancer biomarker that may be useful for the early detection of HNSCC. It is all the more important since mtDNA mutations were reported to be easily detectable in bodily fluids and serum of cancer patients (Fliss *et al*, 2000; Hibi *et al*, 2001a; Jeronimo *et al*, 2001; Parrella *et al*, 2001; Nomoto *et al*, 2002). As a consequence, it would be interesting to search for D-Loop mutations in saliva and serum of a large series of HNSCC patients in order to evaluate their relevance in association with other tumour-specific molecular alterations in the screening of this cancer in alcohol and tobacco consumers.

We analysed for the first time the correlation between mtDNA mutations and clinicopathologic characteristics in a series of HNSCC patients. We found that tobacco consumption was significantly more important in the group of patients with tumour D-Loop mutation than in those without mutation. It is not very surprising since cigarette smoke contains several mutagenic ROS-forming subtances and carcinogens that can cause DNA damages, especially in mtDNA that has been proved to be more susceptible to oxidative damages than nDNA (Marcelino and Thilly, 1999). In the same way, mtDNA content alterations have been recently shown to be associated with smoking (Ballinger *et al*, 1996; Lee *et al*, 1998). Our results also show an association between tumour site and D-Loop mutation, demonstrating that hypopharyngeal tumours were significantly more frequent in the group of mutated tumours compared to that of nonmutated tumours. This association remains unclear. One explanation could be a difference in exposition degree to mtDNA mutagenic agents between hypopharynx and other tumour sites. It was shown a significant higher risk for cancer of the hypopharynx than for larynx cancer with alcohol drinking, which may be explained by the fact that hypopharynx enters in contact with the bolus (alcohol) and the air (tobacco smoke) while air pass through larynx but not the bolus (Esteve *et al*, 1996; Menvielle *et al*, 2004). Alcohol is known to cause oxidative stress through production of ROS (Hoek *et al*, 2002) and ethanol consumption has been reported to induce oxidative damage to mtDNA with increased levels of 8-hydroxydeoxyguanosin in rats (Cahill *et al*, 1997). The abasic sites and oxidised bases generated could so be responsible for mtDNA mutations. We did not found any correlation between D-Loop mutation and gender. This may be explained by the disequilibrium between the group of male (*n*=98) and female (*n*=11) subjects. A better distribution between gender would have been desirable but is not possible to obtain in practice since the sex ratio currently observed in France for HNSCC is approximately M:7/F:1.

In the present study, our first intention was not to investigate the prognostic value of D-Loop mutations and their association with response to chemotherapy, but to evaluate their role as a biomarker of HNSCC by determining the real frequency in HNSCC and their potential correlation with clinicopathologic parameters. However, neoadjuvant chemotherapy has been recently developed in locally advanced HNSCC because conventional treatments with radiotherapy and surgery do not always control them, are associated with profound functional morbidity and often result in physical and psychological suffering. Cisplatin and 5FU combination, which is the standard regimen in this case, has shown objective response rates of 71%, with complete response rates of 18% (Altundag *et al*, 2005). This neoadjuvant chemotherapy do not improve survival but is of interest in terms of organ preservation strategies in responder patients (Laccourreye *et al*, 1999) and has been shown to be curative in complete clinical responder patients with a cancer of the pharyngolarynx (Laccourreye *et al*, 2001). Therefore, identification of factors linked to response to neoadjuvant chemotherapy in HNSSC may help in the selection of patients who could benefit from an organ preservation. Some studies have revealed that the drug-resistance phenotype can be significantly associated with resistance to apoptosis induced by drugs (Landowski *et al*, 1997). Mitochondria is known to play a pivotal role in apoptosis (Green and Kroemer, 2004), and it was suggested that mtDNA determines the cellular response to some cancer therapeutic agents through a mechanism that could be an apoptosis modulation (Singh *et al*, 1999; Hail *et al*, 2001). We recently found that D-Loop mutations were associated with poor prognosis and absence of benefit from adjuvant 5FU chemotherapy in colon cancers (Lievre *et al*, 2005). Given these results, as all patients in our study received a neoadjuvant chemotherapy with 5FU–cisplatin, we investigated the association between D-Loop mutations and response to this treatment. No association with prognosis or with response to 5FU-cisplatin was found. These results suggest that mtDNA do not play the same role in the cytotoxicity of these two anticancer drugs. As 5FU interferes with the apoptotic process (Sakaguchi *et al*, 1994), we hypothesised in our previous study (Lievre *et al*, 2005) that the potential respiratory chain alteration induced by D-Loop mutations could explain in part the absence of benefit from 5FU we observed. On the contrary, Liang *et al* demonstrated that mtDNA depletion increased the sensitivity of cisplatin–induced apoptosis in U937 cells when compared to parental controls containing mtDNA (Liang and Ullyatt, 1998). According to these findings, we can speculate that D-Loop mutations may lead both to resistance to 5FU and increased sensitivity to cisplatin, which may explain that no link was observed between these mutations and response to the combined therapy in the present study. However, the power of our study remains low as regards to the small sample size and the unequal distribution of patients into the two groups considered for the analysis (23 tumours with D-Loop mutations, 86 tumours without), and larger studies are needed to confirm this result.

In conclusion, our data suggest that D-Loop mutations should be considered as a cancer biomarker that may be useful for the early detection of HNSCC in individuals at risk of this cancer. The presence of these mutations in saliva and serum of tobacco and alcohol consumers should be investigated in further studies in order to evaluate their relevance in the screening of these cancers in association with other tumour-specific molecular alterations.

## Figures and Tables

**Table 1:** Clinicopathologic characteristics of HNSCC patients according to D-Loop mutation

**Clinicopathologic characteristics**	**Number of patients**	**Patients with mutated tumour (%)**	**Patients with nonmutated tumour (%)**	***P*-value**
Age (mean)		56.9±2	58.0±1	0.655
*Gender*				
Male	98 (89.9)	22 (95.6)	76 (88.4)	0.3
Female	11 (10.1)	1 (4.4)	10 (11.6)	
				
*Tumour sites*				
Oral cavity	13 (11.9)	1 (4.4)	12 (13.9)	0.03
Oropharynx	46 (42.2)	7 (30.4)	39 (45.4)	
Hypopharynx	27 (24.8)	11 (47.8)	16 (18.6)	
Endolarynx	23 (21.1)	4 (17.4)	19 (22.1)	
				
*Tumour stage*				
I	3 (2.8)	2 (8.7)	1 (1.2)	0.24
II	23 (21.1)	4 (17.4)	19 (22.1)	
III	25 (22.9)	6 (26.1)	19 (22.1)	
IV	58 (53.2)	11 (47.8)	47 (54.6)	
				
Tobacco (mean pack-year)		48±3	37±2	0.01
				
*TP53 mutation*				
Yes	72 (67.3)	19 (82.6)	53 (63.1)	0.07
No	35 (32.7)	4 (17.4)	31 (36.9)	
				
*Response to chemotherapy*				
Responder	72 (66.1)	14 (60.9)	58 (67.4)	0.55
Nonresponder	37 (33.9)	9 (39.1)	28 (32.6)	
Total	109 (100)	23	86	

HNSCC=head and neck squamous cell carcinoma; D-Loop=Displacement-Loop.

**Table 2:** Summary of D-Loop somatic mutations found in the 109 HNSCC patients

**Patients number**	**Nucleotide position**	**DNA (N → T)^a^**	**MtDNA mutation status**
16	303–309	C7 → C7/C9	Heteroplasmy
22	214	A → G	Homoplasmy
	303–309	C8/C9 → C8	Homoplasmy
23	514	(CA)5 → (CA)4	Homoplasmy
28	314	C → T	Homoplasmy
32	303–309	C8 → C7	Homoplasmy
42	303–309	C7 → C7/C9	Heteroplasmy
43	303–309	C8/C9 → C8/C10	Heteroplasmy
48	303–309	C8 → C7/C8	Heteroplasmy
53	303–309	C8 → C8/C9	Heteroplasmy
63	303–309	C8/C9 → C8	Homoplasmy
82	303–309	C9/C10 → C9	Homoplasmy
110	303–309	C7 → C6/C7	Heteroplasmy
114	303–309	C8/C9 → C9/C10	Heteroplasmy
118	213	A → G	Homoplasmy
127	303–309	C8 → C8/C9	Heteroplasmy
141	303–309	C9 → C8	Homoplasmy
146	408	T → A	Heteroplasmy
153	239	C → T	Homoplasmy
	303–309	C7 → C9/C10	Heteroplasmy
155	303–309	C8/C9 → C9	Heteroplasmy
161	303–309	C8 → C7/C8	Heteroplasmy
167	303–309	C8/C9 → complex	Heteroplasmy
180	303–309	C9 → C8/C9	Heteroplasmy
206	303–309	C8 → C8/C9	Heteroplasmy

a N=normal tissue; T=tumour tissue.

HNSCC=head and neck squamous cell carcinoma; D-Loop=Displacement-Loop.

**Table 3:** Summary of the germline polymorphisms found in the 109 head and neck squamous cell carcinoma patients

**Patients number**	**Nucleotide substitution**	**Nucleotide position**	**Reported polymorphism (database^a^)**
16, 53	G → A	200	Yes
20, 48, 206	T → C	204	Yes
48, 206	G → A	207	Yes
27, 32, 52, 128, 150	G → A	228	Yes
82	T → C	239	Yes
199	A → G	240	Novel
41, 54, 171	C → T	242	Yes
133	A → G	249	Novel
48	T → C	250	Yes
44	A → G	257	Yes
171	C → T	295	Yes
9, 13, 18, 26, 31, 35, 65, 72, 76, 84, 87, 88, 116, 164, 169, 177, 183, 190	C7 → C8	303–309	Yes
20, 133, 156, 159	C7 → C9	303–309	Yes
22	C → T	456	Yes
28, 32, 41, 52, 54, 128, 171	C → T	462	Yes
44, 119	T → C	477	Yes
28, 32, 41, 52, 54, 128, 171, 172	T → C	489	Yes
26, 83, 194, 208	C → T	497	Yes
47	A → C	512	Yes
23, 32, 71, 72, 83, 130, 146, 172, 195	(CA)5 → (CA)4	514	Yes
192	(CA)5 → (CA)6	514	Yes
9, 22	(CA)5 → (CA)7	514	Yes
65	A → G	533	Yes
165	C → T	534	Novel

a MITOMAP database available on web: www.mitomap.org.
